# Human Cytomegalovirus Drives Epigenetic Imprinting of the *IFNG* Locus in NKG2C^hi^ Natural Killer Cells

**DOI:** 10.1371/journal.ppat.1004441

**Published:** 2014-10-16

**Authors:** Merlin Luetke-Eversloh, Quirin Hammer, Pawel Durek, Karl Nordström, Gilles Gasparoni, Matthias Pink, Alf Hamann, Jörn Walter, Hyun-Dong Chang, Jun Dong, Chiara Romagnani

**Affiliations:** 1 Innate Immunity, Deutsches Rheuma-Forschungszentrum - A Leibniz Institute, Berlin, Germany; 2 Experimental Rheumatology, Deutsches Rheuma-Forschungszentrum - A Leibniz Institute, Berlin, Germany; 3 Cell Biology, Deutsches Rheuma-Forschungszentrum - A Leibniz Institute, Berlin, Germany; 4 Department of Genetics, University of Saarland, Saarbrücken, Germany; La Jolla Institute for Allergy and Immunology, United States of America

## Abstract

Memory type 1 T helper (T_H_1) cells are characterized by the stable expression of interferon (IFN)-γ as well as by the epigenetic imprinting of the *IFNG* locus. Among innate cells, NK cells play a crucial role in the defense against cytomegalovirus (CMV) and represent the main source of IFN-γ. Recently, it was shown that memory-like features can be observed in NK cell subsets after CMV infection. However, the molecular mechanisms underlying NK cell adaptive properties have not been completely defined. In the present study, we demonstrated that only NKG2C^hi^ NK cells expanded in human CMV (HCMV) seropositive individuals underwent epigenetic remodeling of the *IFNG* conserved non-coding sequence (CNS) 1, similar to memory CD8^+^ T cells or T_H_1 cells. The accessibility of the CNS1 was required to enhance IFN-γ transcriptional activity in response to NKG2C and 2B4 engagement, which led to consistent IFN-γ production in NKG2C^hi^ NK cells. Thus, our data identify epigenetic imprinting of the *IFNG* locus as selective hallmark and crucial mechanism driving strong and stable IFN-γ expression in HCMV-specific NK cell expansions, providing a molecular basis for the regulation of adaptive features in innate cells.

## Author Summary

Upon viral infection, the innate interferon (IFN)-γ producing Natural Killer (NK) cells provide fast, but short-term protection, while adaptive T cells confer delayed, but long-lasting immunity. Once acquired, effector properties remain stably imprinted in the T cell memory progeny. Recently, it was shown that human cytomegalovirus (HCMV) infection can shape the human NK cell repertoire and drive the generation and maintenance of NK cell expansions, which express the activating receptor CD94/NKG2C and have been described as memory-like NK cells. However, the molecular mechanisms underlying NK cell adaptive properties driven by HCMV infection have not been completely defined. In this study, we identify epigenetic imprinting of the *IFNG* locus as selective hallmark and crucial mechanism driving strong and stable IFN-γ expression in HCMV-specific NK cell expansions, thus providing a molecular basis for the regulation of adaptive features in innate cells.

## Introduction

In order to successfully fight infections caused by intracellular pathogens, interferon (IFN)-γ is expressed during an immune response primarily by T cell lineages and natural killer (NK) cells. While NK cells display constitutive *IFNG* promoter activity and express IFN-γ at early maturation stages [1], the expression by CD8^+^ and CD4^+^ T cells is restricted to differentiated effector/memory cells. In particular, naïve CD4^+^ T cells must undergo a differentiation process towards type 1 T helper cells (T_H_1), in order to acquire the ability to stably express IFN-γ [2], [3]. A key mechanism stabilizing T_H_1-lineage commitment is epigenetic imprinting of the *IFNG* locus, which leads to heritable DNA and histone modifications of *cis*-elements, such as the promoter and several conserved non-coding sequences (CNS). The *IFNG cis*-elements that show the most stringent T_H_1-specific hypersensitivity include the promoter and the activation-induced proximal upstream element CNS1, which is located 6 kb or 4 kb upstream of the mouse *Ifng* and human *IFNG* promoter, respectively. These regulatory regions display binding sites for T-bet, STAT4, NF-κB, and NFAT. Once in an open configuration, both regions function as crucial enhancers of *IFNG* transcriptional activity in T_H_1 cells, especially in response to TCR stimulation, due to the presence of binding sites for NFAT, which is activated after engagement of the TCR but not of the cytokine receptors in T cells [3]–[8]. Moreover, effector/memory but not naïve CD8^+^ T cells were shown to display an open configuration in these two *IFNG* regions [9], [10]. Although epigenetic analysis of the *IFNG* locus in total NK cells revealed an open configuration in some regions analyzed [1], [9], [11]–[13], the *IFNG* promoter undergoes partial demethylation during NK cell differentiation [14]. Thus, a more detailed analysis of additional *IFNG* regulatory regions in NK cell subsets is of great importance.

Though being part of the innate immune system, defined subsets of NK cells display adaptive features [15]. NK cells with memory-like properties have been described during mouse cytomegalovirus (MCMV) infection [16], after hapten-induced contact hypersensitivity [17], as well as after cytokine (IL-15+IL-12+IL-18) priming [18]–[20]. Primary MCMV infection of C57BL/6 mice induces the clonal expansion of Ly49H^+^ NK cells, which can directly interact with the m157 protein of MCMV [21]. Ly49H^+^ memory-like NK cells persist for several months and, upon rechallenge, undergo secondary expansion and display enhanced effector functions [16]. The mechanisms underlying the generation of memory-like features in NK cells are a field of intense research [22]–[27]. For instance, it has been shown that proliferation of MCMV-specific Ly49H^+^ NK cells relies on IL-12R and STAT4 signaling and PLZP-mediated antagonism of Blimp-1 [22], [24]. However, it is not clear whether this phenomenon might be associated with imprinting of the *IFNG* locus, similar to memory CD8^+^ T cells and CD4^+^ T_H_1 cells. Human CMV (HCMV) infection also plays an important role in shaping the human NK cell repertoire. In HCMV seropositive (HCMV^+^) individuals, CD94/NKG2C^+^, further referred to as NKG2C^+^, NK cells are present at higher frequency compared to HCMV seronegative (HCMV^−^) ones [28] and can be expanded in vitro in the presence of HCMV-infected fibroblasts [29]. In the course of HCMV reactivation after solid organ or hematopoietic cell transplantation, NKG2C^+^ NK cells from certain donors undergo extensive proliferation in vivo [30]–[33]. These NKG2C^+^ expansions are characterized by bright expression of NKG2C (NKG2C^hi^) and by a terminally differentiated phenotype, as suggested by the preferential expression of CD57 [31], [34]. The specific mechanisms how HCMV might mediate “priming” and expansion of NKG2C^hi^ NK cells is still not completely clear. Both NKG2C and its inhibitory counterpart CD94/NKG2A, further referred to as NKG2A, recognize the non-classical HLA class I molecule HLA-E [35]–[37], whose surface expression is stabilized by peptides derived from the leader sequence of other HLA class I molecules in a transporter associated with antigen processing (TAP)-dependent fashion [38], [39]. Interestingly, a peptide derived from the gpUL40 protein of HCMV strains and clinical isolates is able to stabilize HLA-E in a TAP-independent manner and enables recognition by both NKG2A and NKG2C [40], [41]. HCMV exploits several strategies to induce down-regulation of MHC class I molecules in infected cells, for instance by blocking TAP [42]. Thus, while classical MHC class I molecules are down-regulated, gpUL40 induces surface expression of HLA-E on infected cells [40]. As the inhibitory receptor NKG2A generally displays a higher affinity for HLA-E compared to NKG2C [41], [43], gpUL40-induced up-regulation of HLA-E may represent an escape route for HCMV against NK cell mediated elimination of infected cells. However, while NKG2C is often co-expressed with NKG2A in HCMV^−^ individuals, NKG2C^hi^ expansions in HCMV^+^ individuals typically lack NKG2A, while expressing killer cell immunoglobulin-like receptor (KIR) specific for self-MHC (sKIR), especially KIR2DL1 or KIR2DL2/3, and LIR-1 [28], [33], [34], [44]. Importantly, NKG2C^hi^ expansions persist over time, are highly competent IFN-γ producers and exhibit even higher IFN-γ expression upon HCMV reactivation [30]–[32]. For all these reasons, it has been suggested that NKG2C^hi^ NK cell expansions found in HCMV infected individuals might also represent NK cells with memory-like properties [15], [31], [32].

With the aim to analyze whether epigenetic regulation of the *IFNG* locus could contribute to determine high IFN-γ competence and stability in NK cells described to display memory-like properties, we here showed that only HCMV-specific NKG2C^hi^ expansions and cytokine-primed NK cells displayed an open configuration at the *IFNG* CNS1, which acted as important enhancer of *IFNG* transcriptional activity in response to NKG2C and activating receptors (actR) engagement.

## Results

### Epigenetic remodeling of the CNS1 is an exclusive marker of NKG2C^hi^ expansions and cytokine-primed NK cells

**Figure 1:**
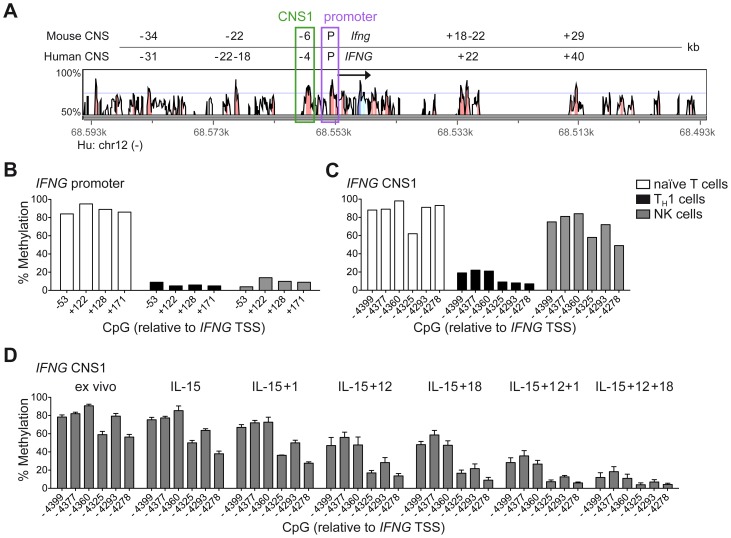
Cytokine-primed NK cells undergo epigenetic remodeling of the *IFNG* CNS1. (A) Alignment of the human *IFNG* and mouse *Ifng* locus as presented by VISTA browser, DNA sequence identity >50% over at least 100 bp. Conserved regions with >70% sequence identity are marked in red and are depicted relative to the *IFNG*/*Ifng* transcriptional start site (TSS). Arrow indicates transcription direction and exon/UTR regions are indicated in blue. (B–D) Methylation status of the *IFNG* promoter and/or CNS1 was analyzed by determining CpG methylation of isolated DNA by bisulfite pyrosequencing. Five CpGs from −53 to +171 bp located in the *IFNG* promoter and six CpGs from −4399 to −4278 bp in the CNS1 region were analyzed and mean percentage of methylation at each individual CpG is depicted. (B and C) CpG methylation of naïve CD4^+^ T cells, T_H_1 cells and NK cells, FACS sorted ex vivo as described in Materials and Methods. One representative experiment out of two (T cells) or out of three (NK cells) is shown. (D) FACS sorted total NK cells were labeled with 500 nM CFSE and cultured in the presence of the indicated cytokines. After five days, viable CFSE^lo^ NK cells, which have undergone proliferation, were FACS sorted and the methylation status of the *IFNG* CNS1 was analyzed. Mean percentage of methylation ± SEM at each individual CpG is depicted (n = 3).

**Figure 2:**
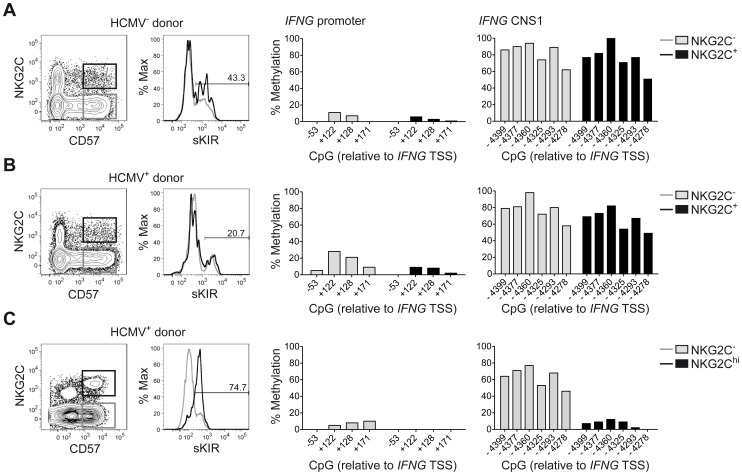
Expanded NKG2C^hi^ NK cells display complete demethylation of the *IFNG* CNS1. Analysis of surface marker expression and methylation status of the *IFNG* promoter and CNS1 was analyzed as described in Figure 1 in ex vivo NK cell subsets derived from representative HCMV^−^ (n = 2) (A) or HCMV^+^ (n = 4) donors (B and C), without (B) or with expansion of NKG2C^hi^ NK cells (C). NKG2C and CD57 expression was analyzed by FC on PBMC, after gating on viable CD3^−^ CD56^dim^ NK cells, while sKIR (KIR2DL3 in HLA-C1^+^ donor) expression was analyzed after gating on viable CD3^−^ CD56^dim^ CD57^+^ NKG2C^+/−^ NK cells. CpG methylation of the *IFNG* promoter and CNS1 was analyzed in FACS sorted CD56^dim^ CD57^+^ NKG2C^−^ (gray bars) and in CD56^dim^ CD57^+^ NKG2C^+/hi^ NK cells (black bars) from each donor and is depicted as mean percentage of methylation at each CpG site.

Among the described regulatory regions conserved between the human and mouse *IFNG/Ifng* locus (Figure 1A), we analyzed the methylation status of selected CpG residues present in the frame of the promoter and the CNS1, which are important enhancers of *IFNG* transcriptional activity and represent epigenetic hallmarks of T_H_1 differentiation [3], [4]. While the promoter was largely demethylated in NK cells and T_H_1 cells (Figure 1B), CNS1 was completely methylated in NK cells, resembling the configuration of naïve CD4^+^ T cells, rather than of T_H_1 cells (Figure 1C). Previous reports have described that after HCMV infection or priming with IL-15+IL-12+IL-18, NK cells can display memory-like properties and the ability to stably produce high amounts of IFN-γ after rechallenge [19], [30], [31]. Considering the parallels to memory T_H_1 cell formation, we analyzed the methylation status of the CNS1 in cytokine-primed NK cells and in HCMV-specific NK cell expansions. Cytokine-primed NK cells generated in vitro in the presence of IL-15+IL-12+IL-18 displayed complete demethylation of the CNS1. Conversely, only partial and progressive opening of both regions could be observed in the presence of IL-15+IL-12, IL-15+IL-18 or IL-15 alone. Substituting IL-18 by IL-1 almost led to similar results (Figure 1D). Next, we analyzed the methylation status of the *IFNG* CNS1 in NKG2C^+^ and NKG2C^−^ NK cells isolated from HCMV^−^ (Figure 2A) or HCMV^+^ (Figure 2B) individuals as well as in NKG2C^+^ NK cell expansions, selectively occurring in some HCMV^+^ individuals (Figure 2C). These NKG2C^+^ expansions are characterized by preferential expression of CD57 and strong enrichment in at least one sKIR (Figure 2C). As CD57^+^ NKG2C^+^ expansions display higher surface expression of NKG2C (Figure S1), they are further referred to as NKG2C^hi^ NK cells [31], [44]. For a fair comparison of all NK cell subsets, CpG methylation analysis was performed after sorting CD56^dim^ CD57^+^ NK cells. CD56^dim^ CD57^+^ NK cells displayed an open configuration of the *IFNG* promoter independent of NKG2C expression or HCMV status (Figures 2A, 2B and 2C). NKG2C^−^ NK cells derived from HCMV^+^ or HCMV^−^ individuals as well as NKG2C^+^ NK cells from HCMV^−^ individuals consistently displayed a closed configuration of the CNS1 (Figures 2A, 2B and 2C). Importantly, among HCMV^+^ individuals, an open configuration of the CNS1 occurred exclusively in donors displaying a distinctive expansion of NKG2C^hi^ sKIR^+^ NK cells (Figure 2C). Conversely, if no expansion was present, NKG2C^+^ NK cells from HCMV^+^ individuals displayed a closed configuration of the CNS1, similar to the NKG2C^−^ subsets (Figure 2B). Altogether, these data show that among NK cells only HCMV-specific NKG2C^hi^ expansions and cytokine-primed NK cells, which were described as memory-like NK cells [15], [19], [30], [31], share the ability to undergo remodeling of the CNS1 with T_H_1 cells.

### CNS1 demethylation occurs in NKG2C^hi^ NK cell expansions, independent of sKIR or CD57 expression and remains stably imprinted in the progeny

**Figure 3:**
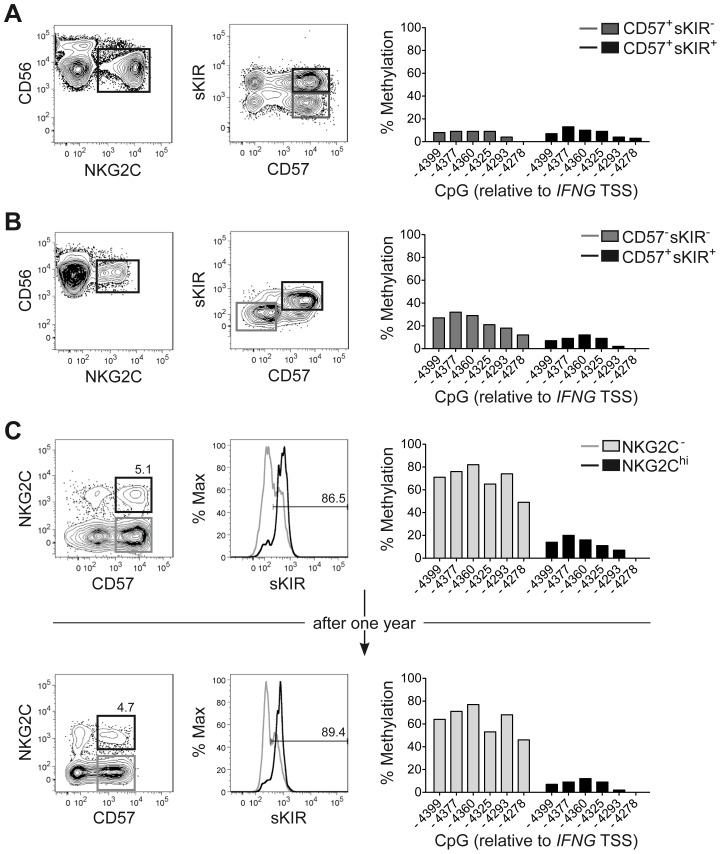
CNS1 demethylation occurs independent of CD57/sKIR expression and is stably imprinted in NKG2C^hi^ NK cells. (A–C) Phenotype and methylation status of the CNS1 was analyzed in ex vivo NKG2C^hi^ NK cell expansions from representative HCMV^+^ donors (n = 4), as described in Figure 1. (A and B) CD56 and NKG2C expression was analyzed by FC on PBMC, after gating on viable CD3^−^ CD56^+^ NK cells, while CD57 and sKIR (KIR2DL3 in HLA-C1^+^ donor) expression was analyzed after gating on CD56^dim^ NKG2C^hi^ NK cells. CpG methylation of the *IFNG* CNS1 was analyzed in FACS sorted CD56^dim^ NKG2C^+^ NK cell subsets as indicated and is depicted as mean percentage of methylation at each CpG site. (C) The same HCMV^+^ individual displaying an expanded NKG2C^hi^ population was analyzed twice with an interval of one year between the two measurements. NKG2C and CD57 expression was analyzed by FC on PBMC, after gating on viable CD3^−^ CD56^dim^ NK cells, while sKIR expression was analyzed after gating on CD56^dim^ CD57^+^ NKG2C^+/−^ NK cells. CpG methylation of the *IFNG* CNS1 was analyzed in FACS sorted CD56^dim^ CD57^+^ NKG2C^−^ (gray bars) and in CD56^dim^ CD57^+^ NKG2C^+^ NK cells (black bars) and is depicted as mean percentage of methylation at each CpG site.

One major phenotypic difference between CD56^dim^ CD57^+^ NKG2C^hi^ cell expansions and CD56^dim^ CD57^+^ NKG2C^+^ cells from HCMV^+^ donors is the high enrichment in expression of at least one sKIR, which defines educated NK cells. Thus, we next asked whether CNS1 demethylation was a peculiar feature of sKIR^+^ educated NK cells, rather than of NKG2C^hi^ cells. Among NKG2C^hi^ NK cells, CNS1 was accessible not only in CD57^+^ sKIR^+^ cells, but also in CD57^+^ sKIR^−^ cells (Figure 3A). Surprisingly, among NKG2C^+^ cells, CNS1 was accessible even in CD57^−^ sKIR^−^ cells (Figure 3B). We finally aimed to determine whether the open configuration of the CNS1 was stably imprinted in NKG2C^hi^ NK cell expansions, by analyzing the same HCMV^+^ individual after one year. The size and phenotype of the NKG2C^hi^ NK cell pool remained constant over time (Figure 3C), in line with previous data [44]. Importantly, CD57^+^ NKG2C^hi^ but not CD57^+^ NKG2C^−^ NK cells still displayed a completely open configuration at both the *IFNG* promoter and CNS1 (Figures 3C and S2), indicating that the *IFNG* locus remains stably imprinted in NKG2C^hi^ NK cell progeny, similar to memory T_H_1 cells.

### NKG2C^hi^ NK cell expansions share a global epigenetic signature with T_H_1 and CD8^+^ memory T cells

**Figure 4:**
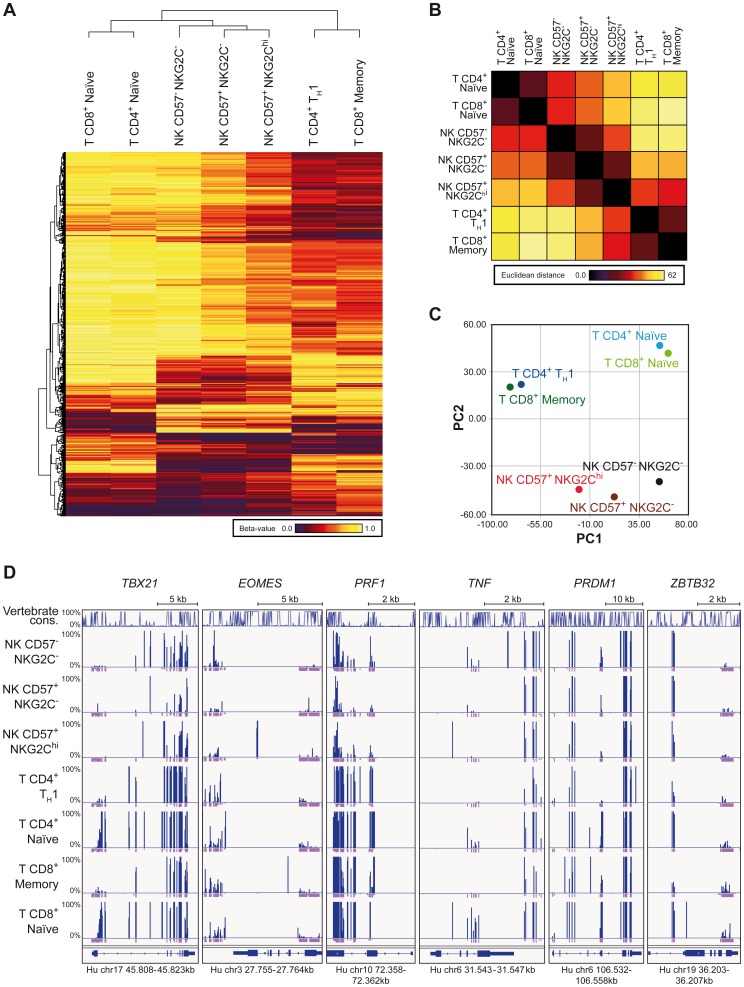
RRBS-based global methylation analysis of NK cell and T cell subsets. Genomic DNA of ex vivo FACS sorted NK and T cell subsets was analyzed for CpG methylation by RRBS. (A–C) Beta-values for each methylation site identified by RRBS were averaged from two donors for each T or NK cell subset as indicated. Sites with coverages below 5 were removed from further analysis. (A) Clustering analysis of differentially methylated CpG sites found by RRBS. Clustering was performed on 1,000 most variant methylation sites out of 30,000 randomly chosen sites, localized within gene bodies and promoter regions. The indicated cell subsets cluster according to the similarities of their methylation profile. Beta-values depicted from dark-red to yellow represent level of methylation of the individual CpG sites. (B) Similarities between the cell subsets as judged by the Euclidian distance. (C) Principle component analysis (PCA) of mean methylation levels from two donors for indicated cell subsets. The PCA revealed a clear separation of CD4^+^ T_H_1 and CD8^+^ memory cells, naïve CD4^+^ and CD8^+^ cells, and NK cells by the first two components. (D) CpG methylation sites of T and NK cell subsets of one representative donor identified by RRBS in gene bodies of *TXB21*, *EOMES*, *PRF1*, *TNF*, *PRDM1* and *ZBTB32*. Line diagram represents sequence conservation within vertebrates. Blue and violet bars indicate the methylation level (0–100%) and coverage for each identification, respectively. Coverages of 5 and more reads are represented as a full violet bar.

In order to understand whether *IFNG* CNS1 demethylation was part of a broader epigenetic remodeling occurring in NKG2C^hi^ expansions from HCMV^+^ individuals, we performed RRBS-based global methylation analysis of CD57^+^ NKG2C^hi^, CD57^+^ NKG2C^−^ and CD57^−^ NKG2C^−^ NK cells in comparison with CD8^+^ memory and CD4^+^ T_H_1 cells as well as with CD8^+^ or CD4^+^ naïve T cells isolated from the same HCMV^+^ individuals. CD8^+^ memory and CD4^+^ T_H_1 cells, CD8^+^ or CD4^+^ naïve T cells, and NK cells constitute three different clusters. Interestingly, NKG2C^hi^ NK cells distinctively shared the methylation profile of a large set of CpG sites with CD8^+^ memory or CD4^+^ T_H_1 cells. Conversely, CD57^−^ NKG2C^−^ NK cells were more similar to naïve CD8^+^ or CD4^+^ T cells, with CD57^+^ NKG2C^−^ NK cells displaying a methylation profile intermediate to the two other NK cell subsets (Figure 4A). The NK cell subsets show a decreasing gradient of similarities to naïve CD8^+^ and CD4^+^ T cells from CD57^−^ NKG2C^−^ via CD57^+^ NKG2C^−^ to CD57^+^ NKG2C^hi^, as judged by the Euclidian distance, and a complementary, increasing gradient of similarities to CD8^+^ memory and CD4^+^ T_H_1 cells (Figure 4B). This observation was further confirmed by employing an unsupervised strategy and performing principal component analysis (PCA) (Figure 4C). Most of the variation among the different lymphocyte subsets was captured by the first principal component (PC1). CD8^+^ and CD4^+^ naïve T cells were at one extreme, while CD8^+^ memory together with T_H_1 cells were at the other extreme of the PC1-axis, suggesting that PC1 defined the direction of lymphocyte differentiation, which was characterized by a global epigenetic remodeling (Figure 4B). NK cell subsets distributed along the PC1, with CD57^−^ NKG2C^−^ cells displaying the highest PC1 score, similar to naïve T cells, while CD57^+^ NKG2C^hi^ NK cells displayed the lowest PC1 score. T cell and NK cell lineage-defining epigenetic modifications were likely described by the second principal component (PC2). Analyzing the methylation pattern of other genes of interest, which are shared between NK cells and T_H_1 or CD8^+^ memory T cells, such as *TBX21* (T-bet), *EOMES* (Eomesodermin), and *PRF1* (perforin), we observed that all NK cell subsets displayed similar or even more pronounced demethylation pattern at several CpG regions compared to T_H_1 or CD8^+^ memory T cells (Figure 4D). *TNF* was mainly demethylated in both CD57^+^ NKG2C^hi^ and CD57^+^ NKG2C^−^ NK cell subsets, similar to T_H_1 or CD8^+^ memory T cells. Recently, it was shown that *PRDM1* (Blimp-1) and *ZBTB32* (also known as PLZP, ROG, FAZF and TZFP), which are not expressed in naïve T cells but are up-regulated in differentiated T cells [45]–[47], regulate the proliferative burst of Ly49H^+^ memory NK cells during MCMV infection [24]. Interestingly, some CpGs of the *PRDM1* and *ZBTB32* genes were consistently demethylated in NKG2C^hi^ NK cells and T_H_1 or memory CD8^+^ T cells, compared to other NK cell subsets or naïve T cells. Altogether, these data suggest that CNS1 demethylation in NKG2C^hi^ expansions from HCMV^+^ individuals is part of a broader epigenetic remodeling, which is partially shared by T_H_1 and memory CD8^+^ T cells.

### NKG2C is a main trigger for IFN-γ production in NKG2C^hi^ NK cells

**Figure 5:**
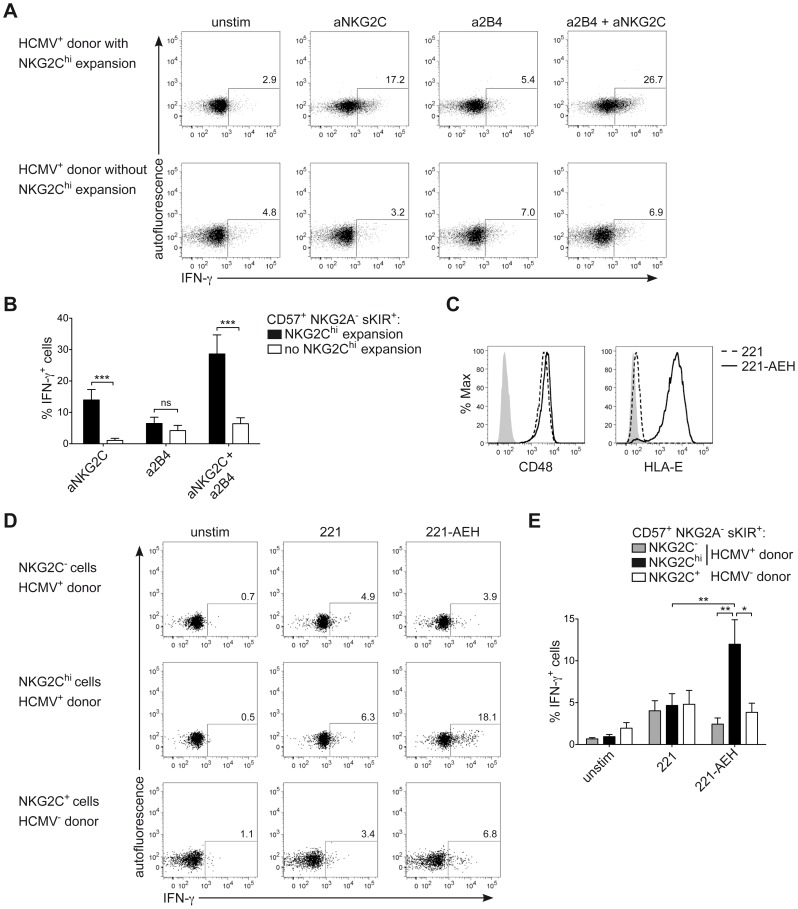
NKG2C^+^ NK cells express IFN-γ in response to NKG2C engagement. (A and B) Viable CD3^−^ CD56^dim^ NK cells were FACS sorted and stimulated by cross-linking of NKG2C and/or 2B4 for 16 hours. Analysis of intracellular IFN-γ expression was performed by FC after gating on CD56^dim^ CD57^+^ NKG2A^−^ sKIR^+^ cells from HCMV^+^ donors displaying or not an expansion of NKG2C^hi^ cells. Gating strategy is depicted in Figure S3D. One representative experiment (A) and mean percentage of IFN-γ producing cells ± SEM (B) are depicted (n≥8). Percentage of IFN-γ producing cells was calculated after subtracting the value observed in unstimulated cells. ***p<0.001 calculated with Mann-Whitney test. (C) Surface expression of CD48 and HLA-E was analyzed by FC on 221 (dashed line) or 221-AEH cells (black line), with isotype control or secondary staining only (solid grey histogram). One representative staining out of two is depicted. (D and E) Viable CD3^−^ CD56^dim^ NK cells were FACS sorted and stimulated by co-culture with 221 or 221-AEH for 6 hours. Analysis of intracellular IFN-γ expression was performed by FC after gating on CD56^dim^ CD57^+^ NKG2A^−^ sKIR^+^ NKG2C^hi/+/−^ cells from HCMV^+/−^ donors. One representative experiment (D) and mean percentage of IFN-γ producing cells ± SEM (E) are depicted (n = 6). *p<0.05, **p<0.01, calculated with Mann-Whitney test.

NKG2C^hi^ NK cells are described as potent IFN-γ producers [30]. However, in line with their phenotype of CD57^+^ terminally differentiated cells, they respond less efficiently to IL-12+IL-18 stimulation, compared to CD57^−^ NK cells, due to the lower expression of *IL12RB2* and IL-18R (Figure S3A and S3B), in line with previous data [34]. Moreover, although terminally differentiated CD57^+^ sKIR^+^ NK cells are generally more proficient in producing IFN-γ in response to activating receptor triggering [14], several actR, such as NKp46 and NKp30 are prominently down-regulated in NKG2C^hi^ NK cells, in line with previous findings [28], [44] and their engagement led to only poor IFN-γ production (Figure S3C and S3E). As NKG2C triggering has been shown to induce effector functions in NKG2C^+^ NK cells from HCMV^+^ donors [31], [34], we hypothesized that NKG2C engagement alone or in combination with 2B4 could be a major trigger of IFN-γ expression in expanded NKG2C^hi^ NK cells. Since NKG2C could not be stained after cross-linking, IFN-γ expression was first analyzed after gating on CD57^+^ NKG2A^−^ sKIR^+^ NK cells, of which the large majority expressed NKG2C only in HCMV^+^ donors displaying an expansion (Figure S3D). Triggering of NKG2C alone was able to induce high IFN-γ production in CD57^+^ NKG2A^−^ sKIR^+^ NK cells only in HCMV^+^ individuals displaying NKG2C^hi^ expansions and this effect was even more pronounced when NKG2C and 2B4 were engaged in combination. Conversely, very low IFN-γ expression could be detected in CD57^+^ NKG2A^−^ sKIR^+^ NK cells from HCMV^+^ individuals with or without NKG2C^hi^ expansions when 2B4 alone was engaged (Figures 5A and 5B). Next, we tested whether a similar effect could be observed when NK cells were stimulated via the physiological engagement of the 2B4 ligand CD48 and the NKG2C ligand HLA-E, by using the CD48^+^ cell line LCL.221 (221) or 221-AEH [39], which expressed high levels of HLA-E on their surface, in contrast to 221 (Figure 5C). In this system, analysis of IFN-γ expression among CD57^+^ NKG2A^−^ sKIR^+^ cells could be performed directly by gating on NKG2C^hi^ or NKG2C^−^ cells from HCMV^+^ donors as well as on NKG2C^+^ cells from HCMV^−^ donors. In line with the data obtained with antibody cross-linking, only NKG2C^hi^ expansions proficiently produced IFN-γ in response to 221-AEH (Figure 5D and 5E). Conversely, stimulation with 221 resulted in only low IFN-γ expression in all subsets analyzed. Thus, these data show that engagement of NKG2C alone or together with 2B4 represents a major trigger of IFN-γ production in NKG2C^hi^ NK cells.

### CNS1 accessibility enhances *IFNG* transcriptional activity induced by NKG2C and 2B4 engagement

**Figure 6:**
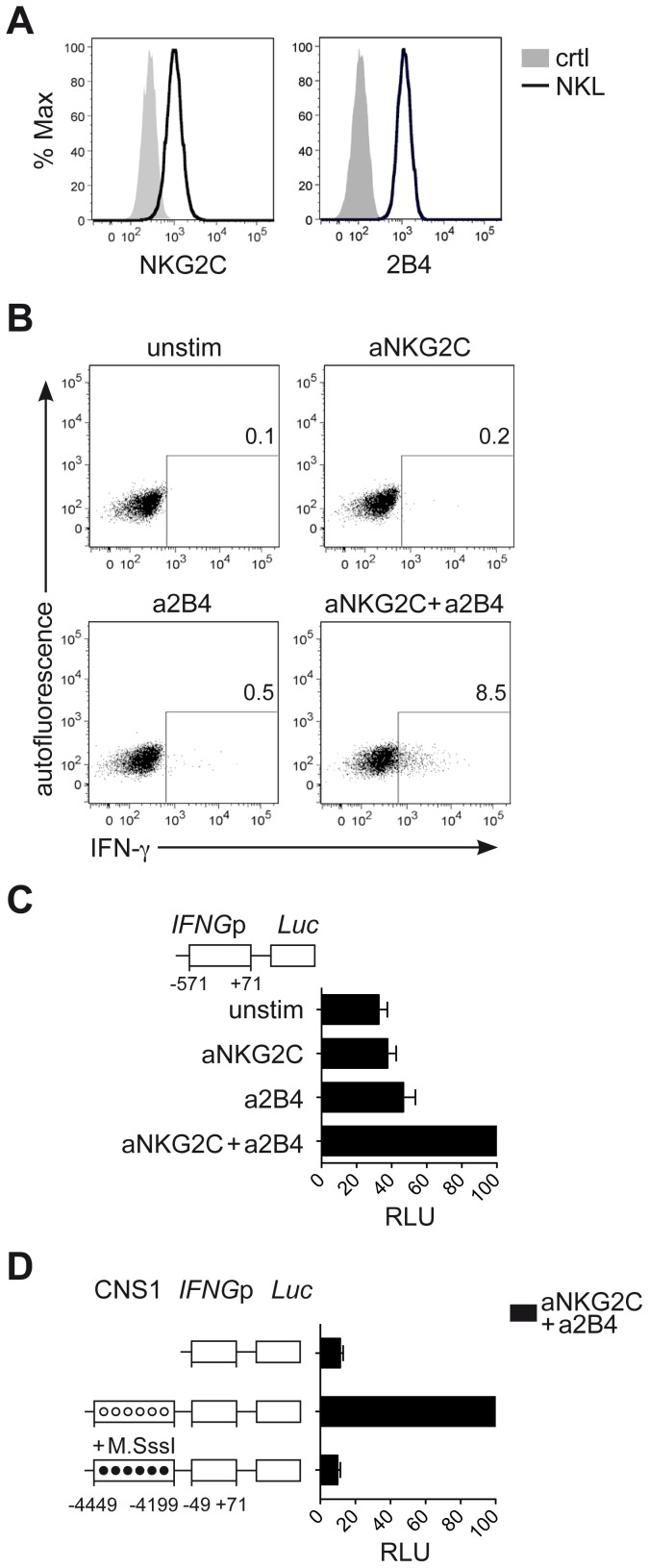
CNS1 accessibility regulates *IFNG* transcriptional activity induced by NKG2C engagement. (A) Surface expression of NKG2C and 2B4 on NKL (black line) or isotype control (solid grey histogram) was determined by FC. (B) Intracellular expression of IFN-γ by NKL was detected by FC after crosslinking of NKG2C and/or 2B4 for 16 hours. One representative experiment out of four is depicted. (C and D) Luciferase reporter assay of *IFNG* sequences transfected in NKL. (C) Construct containing the *IFNG* promoter (*IFNG*p) region was cloned into the Luciferase reporter vector pGL3 upstream of the Firefly luciferase gene (*Luc*). pGL3 reporter vectors were transfected into NKL cells along with Renilla reporter vector pRL-TK as internal control and luciferase activity was measured after stimulation of NKL, as indicated. Relative luciferase units (RLU) were calculated in relation to the activity of the *IFNG*p (−571 to +71) stimulated with aNKG2C+a2B4, after normalization to Renilla luciferase and basic pGL3 activity. Mean RLU ± SEM (n = 3) are depicted. (D) Constructs containing the *IFNG*p (−49 to +71) region with or without the CNS1 were cloned into the CpG-free vector pCpGL. Luciferase activity of untreated (unmethylated, open circles) and of CpG-methyltransferase (M.SssI)-treated vectors (methylated, black circles) was measured after transfection into NKL cells. RLU were calculated relative to the activity displayed by the unmethylated CNS1+*IFNG*p (−49 to +71) vector stimulated with aNKG2C+a2B4, after normalization to Renilla luciferase and basic pCpGL activity. One representative experiment out of three is depicted.

Next, we aimed to understand whether CNS1, which was selectively demethylated in NKG2C^hi^ NK cells, would be able to enhance *IFNG* transcriptional activity induced by engagement of NKG2C alone or in combination with 2B4. To perform luciferase reporter assays, we took advantage of the NK cell line NKL. NKL expressed 2B4 but only intermediate levels of NKG2C (Figure 6A), and only co-engagement of NKG2C and 2B4, but not of NKG2C alone, efficiently induced IFN-γ expression in NKL (Figure 6B). In line with these data, *IFNG* transcriptional activity in NKL, measured after transfection with the luciferase reporter vector pGL3 containing the *IFNG* promoter (*IFNG*p) (−571 to +71), could be induced only after co-engagement of 2B4 and NKG2C (Figure 6C). Next, we tested whether CNS1 was able to enhance *IFNG* transcriptional activity induced by the engagement of NKG2C and 2B4 and whether selective methylation would suppress it. To this aim, we cloned the minimal *IFNG*p (−49 to +71), not containing any CpG site, into the CpG-free luciferase reporter vector pCpGL [48], in combination or without the CNS1 sequence containing the CpG sites of interest. The construct was in vitro treated or not with CpG-methyltransferase (M.SssI), which selectively methylated the CNS1 CpG sites. CNS1 induced a dramatic enhancement of *IFNG* transcriptional activity in response to co-engagement of 2B4 and NKG2C, which was completely abolished by in vitro methylation of the CNS1 construct (Figure 6D). In further support of the concept that DNA methylation plays an important role in IFN-γ expression in NK cells as in T_H_1 cells, we treated NKL with the DNA methyltransferase inhibitor 5-azacytidine (AZA). Addition of AZA resulted in partial CNS1 demethylation and consistently enhanced expression of IFN-γ induced by NKG2C alone or by NKG2C and 2B4 co-stimulation (Figure S4A and S4B). Altogether, this data show that accessibility of CNS1 is crucial to enhance *IFNG* transcriptional activity in response to NKG2C engagement.

## Discussion

Our present study showed that epigenetic remodeling of the CNS1 region in the *IFNG* locus is a hallmark of NKG2C^hi^ NK cells expanded in HCMV^+^ individuals as well as cytokine-primed NK cells, which have been previously described as memory-like NK cells [15], [19], [31], [32] and share an open configuration of this region with T_H_1 cells. CNS1 is a crucial enhancer of *IFNG* transcriptional activity in T_H_1 cells, especially in response to TCR triggering [4], [8]. In line with its important role as TCR-dependent enhancer in T_H_1 cells, we showed that accessibility of CNS1 enhanced *IFNG* transcriptional activity of NK cells upon triggering of actR, especially NKG2C. Whereas resting NK cells generally need simultaneous engagement of multiple actR [49], the sole engagement of NKG2C was sufficient to induce IFN-γ production in expanded NKG2C^hi^ cells, while 2B4 mainly functions as coreceptor [50]. We propose that CNS1 accessibility contributes to the robust IFN-γ expression in response to NKG2C engagement as one important mechanism. Epigenetic remodeling of the *IFNG* locus, together with high expression of NKG2C, might help NK cells to compensate for other actR deficiencies. Several characteristics displayed by NKG2C^hi^ NK cells, such as their in vivo expansion, persistence in time and high effector functions after HCMV reactivation support the concept that these cells display adaptive properties [15]. Indeed, our genome wide analysis of DNA methylation showed that NKG2C^hi^ NK cell expansions show remarkably lower epigenetic distance to T_H_1 and CD8^+^ memory T cells as compared to the other NK cell subsets analyzed. This observation further strengthens the idea that common epigenomic features might be functional hallmarks of memory-like NK cells and memory T cells.

It is still not clear to which extent some of the adaptive features of NKG2C^hi^ expansions depend on low-level viral reactivation events occurring in healthy HCMV^+^ individuals [51]. In line with this interpretation, it was shown that during allogeneic hematopoietic stem cell transplantation from HCMV^+^ donor to HCMV^+^ recipient, NKG2C^+^ NK cells also expand in the absence of clinically detectable HCMV viremia [32]. Similarly, clinically silent reactivation of HCMV or of other herpesviruses also stimulates HCMV-specific T cells and dramatically shapes the T cell repertoire [52], [53].

Which signals are driving CNS1 demethylation exclusively in expanded NKG2C^hi^ NK cells? Co-culture with 221-AEH cells, which express the NKG2C ligand HLA-E, or with HCMV-infected fibroblasts leads to expansion of NKG2C^+^ NK cells [29], [44]. Although engagement of NKG2C might contribute to induce CNS1 opening in these cells, we do not have evidences for that. On the other hand, we show that IL-12+IL-15+IL-18 stimulation is sufficient to drive CNS1 demethylation in NK cells. Priming by pro-inflammatory cytokines, which largely contribute to anti-viral defense [54], might not only induce CNS1 opening but also contribute to select NKG2C^+^ cells with proliferative and/or survival advantage [34], [55], thus enabling the persistence of a progeny, which has undergone epigenetic remodeling of the CNS1, similar to memory T_H_1 cells. Further supporting a central role of pro-inflammatory cytokines in this process, it was shown that generation of MCMV-specific Ly49H^+^ memory-like NK cells is strongly dependent on IL-12R/STAT4 signaling [22] and that induction of PLZP, required for the proliferative burst of Ly49H^+^ NK cells, is accomplished by IL-12 and IL-18 but not by Ly49H engagement [24]. A preferential expansion/survival in response to inflammatory cytokines might also partially explain why NKG2C^+^ NK cells from HCMV^+^ individuals could expand in the course of other viral infections, such as with Hantavirus, Chikungunya or Hepatitis C virus [34], [55], [56]. This unique ability of NKG2C^hi^ NK cells to expand and become activated in different infections closely resembles what has been observed for HCMV-specific T cell responses. It was shown that the broad HCMV-specific T cell compartment not only controls viral reactivation, but can also alter the outcome of unrelated infections [57], a phenomenon known as heterologous immunity [58]. Herpesvirus-specific CD8^+^ T cells, especially HCMV- and EBV-specific ones, are frequently activated in the setting of other infections, among which Hantavirus and Hepatitis virus have been reported [57], [59]. This phenomenon does not rely exclusively on T cell receptor cross-reactivity but also acts through antigen-independent bystander mechanisms, such as cytokines, in particular IL-15 [51], [57]. Similarly, during Hantavirus and Hepatitis virus infections proinflammatory cytokines might promote the expansion of NKG2C^hi^ NK cells, which were previously primed during HCMV infection. Interestingly, we also showed that, among CD56^dim^ NKG2C^hi^ NK cells, CNS1 demethylation is not confined to the CD57^+^, but also present in the CD57^−^ subset, which is highly responsive and proliferates in response to cytokines [44], [60]. Thus, our and other data [44] suggest that CD57^−^ NKG2C^hi^ cells, rather than their CD57^+^ counterpart, might represent memory-like precursors replenishing and stably maintaining the peripheral pool of NKG2C^hi^ NK cells.

In summary our data identified epigenetic imprinting in the CNS1 region of the *IFNG* locus as a hallmark and mechanism underlying adaptive features and IFN-γ memory in NK cells. We also showed that a broader epigenetic remodeling accompanies these adaptive features demonstrating that both inflammatory and pathogen cues induce epigenetic remodeling of NK cells. These two observations extend our view of the molecular control caused by inflammation in innate immune cells [61], [62] and furthermore highlight adaptive epigenetic similarities among the two branches of the immune system.

## Materials and Methods

### Ethics statement

Leukocyte concentrates were obtained from adult healthy individuals (DRK, Berlin, Germany) after written informed consent and approval by the local ethics committees on human studies (Charité Berlin, Germany).

### Cell isolation and flow cytometry

Peripheral blood mononuclear cells (PBMC) were isolated by Ficoll Hypaque density gradient centrifugation from leukocyte concentrates obtained from healthy individuals (DRK, Berlin, Germany). NK cells, CD4^+^ and CD8^+^ T cells were enriched by magnetic cell sorting using CD56, CD4 or CD3 microbeads respectively (Miltenyi Biotec), followed by flow cytometric sorting (FACS) using FACS Aria II (BD Biosciences). NK cell subsets were FACS sorted as viable CD3^−^ CD56^+^ (Figure 1) or as CD3^−^ CD56^dim^ CD57^+/−^ (CD62L^+/−^) sKIR^+/−^ NKG2C^+/−^, hereby showing purity over 95%. sKIR corresponds to KIR2DL1^+^ in HLA-C2^+^ or KIR2DL3^+^ in HLA-C1^+^ donors, respectively. Naïve CD4^+^ T cells were FACS sorted as CD3^+^ CD4^+^ CD45RA^+^ CCR7^+^ and T_H_1 cells as CD3^+^ CD4^+^ CD45RA^−^ CCR7^−^ CCR5^+^ IL-18Ra^+^ [63], [64]. Naïve CD8^+^ T cells were FACS sorted as CD3^+^ CD8^+^ CD45RA^+^ CCR7^+^ cells and CD8^+^ memory cells as CD3^+^ CD8^+^ CD45RO^+^ cells. T cell subsets were FACS sorted to over 95% purity. For flow cytometric (FC) analysis, PBMC were stained with mAbs against the following molecules: CD56 PE-Cy7 (NCAM16.2), CD3 APC-H7 (SK27), CD3 V500 (UCHT1), CD4 V450 (RPA-T4), CCR5 APC-Cy7 (2D7/CCR5) (BD Biosciences), CD56 BV605 (HCD56), CD62L PerCP-Cy5.5 (Dreg56), CD57 Pacific Blue (HCD57), CD45RA PE-Cy7 (HI100), CCR7 PerCP-Cy5.5 (G043H7), KIR3DL1 PE (DX9), HLA-E PE (3D12) (Biolegend), KIR2DL1 APC (143211), KIR2DL3 FITC (180701), NKG2C PE (134591), IL-18Ra PE (70625), NKG2C (134522) in-house coupled to biotin (R&D Systems), 2B4 biotin (C1.7) (eBioscience). KIR2DL1/S1 (11PB6) and KIR2DL2/L3/S2 (GL183), kindly provided by D. Pende and A. Moretta, Genova, Italy, were self-conjugated to Cy5 or Alexa Fluor 700. CD8 Cy5 (GN11/134D7) and CD45RO FITC (UCHL1) were self-conjugated. CD48 (CO202) was kindly provided by A. Moretta, Genova, Italy. Secondary staining of biotinylated mAbs was performed with Streptavidin-BV421 (Biolegend), and of unconjugated IgM mAbs with aIgM PE (RMM-1, Biolegend). Viable cells were detected using Live/Dead (LD) fixable dead cell stains (Invitrogen). Flow cytometric (FC) analysis was performed at BD FACS Fortessa employing FACSDiva Software (BD Biosciences). Obtained data was analyzed with FlowJo software (Tree Star).

### Cell culture, stimulation and intracellular staining

FACS sorted NK cell subsets were maintained in RPMI-1640 (Gibco BRL) (100 U/mL penicillin and 0.1 mg/ml streptomycin added) supplemented with 10% human AB serum or 10% FCS (Lonza). To cross-link distinct actR, biotinylated mAbs specific for 2B4 (C1.7, eBioscience), NKp30 (AF29-4D12), NKp46 (9E2) (Miltenyi Biotec) and purified NKG2C (134522, R&D Systems) (in-house coupled to biotin) were conjugated to anti-biotin MACSiBead particles (all Miltenyi Biotec). For NK cell stimulation, beads were used at a bead∶cell ratio of 5∶1 and co-incubated with NK cells for 16 hours. Alternatively NK cells were stimulated with 50 ng/ml IL-12 and 50 ng/ml IL-18 for 16 hours. 10 µg/ml Brefeldin A (Sigma) was added for the time of stimulation. The target cell line LCL.221 or 221-AEH [39] was used for stimulation at a NK cell∶target cell ratio of 10∶1 for 6 hours, adding 10 µg/ml Brefeldin A and Golgistop (BD Biosciences) after 1 hour. 221 and 221-AEH were maintained in complete RPMI medium supplemented with 10% FCS and 721.221-AEH in the presence of hygromycin B (Invitrogen) at 250 µg/ml. After stimulation, cells were fixed with 1.6% paraformaldehyde (Electron Microscopy Services) and stained intracellularly in the presence of 0.5% saponin (Sigma-Aldrich). Intracellular cytokines were stained using the following mAbs: IFN-γ APC (B27) (BD Biosciences) or IFN-γ PE-Cy7 (B27) (Biolegend). To generate cytokine-induced memory-like NK cells, FACS sorted and CFSE-labeled (500 nM, Invitrogen) NK cells were incubated with 20 ng/ml IL-1β, 10 ng/ml IL-12, 20 ng/ml IL-15 (Miltenyi Biotec) and/or 20 ng/ml IL-18 (R&D systems) for 5 days. The NK cell line NKL (ATCC) was cultured in complete RPMI medium supplemented with 10% FCS in the presence of 100 U/ml IL-2 (Miltenyi Biotec). To test the impact of 5-Azacytidine (AZA), NKL cells were cultured for 5 days in the presence of 5 µM AZA (Sigma).

### Quantitative RT-PCR (qPCR)

RNA was isolated from FACS sorted NK cell subsets using NucleoSpin RNAII (Macherey Nagel) and reverse transcribed using Reverse Transcription Reagents. mRNA levels were analyzed by qPCR using a StepOnePlus real-time PCR system and TaqMan gene expression assays according to manufacturer's instructions (all Applied Biosystems): Hs00155486_m1 *IL12RB2*, 4333764F *GAPDH*. mRNA content was normalized to *GAPDH* expression and mean relative gene expression was determined using the ΔΔCT method.

### CpG methylation analysis

Genomic DNA of ex vivo FACS sorted NK and T cell subsets was extracted using the QIAamp DNA Mini Kit and bisulfite conversion was performed by using the EpiTect Bisulfite Kit (all purchased from Qiagen). Regions of interest in the *IFNG* locus were amplified using primers as described before [9], and subsequently pyrosequenced by Varionostic GmbH, Germany.

### Reduced Representation Bisulfite Sequencing (RRBS)

Genomic DNA of ex vivo FACS sorted CD3^−^ CD56^dim^ CD57^+/−^ NKG2C^+/−^ NK cell subsets, naïve CD4^+^ T cells, T_H_1 cells as well as naïve and memory CD8^+^ T cells from two donors was extracted using the QIAamp DNA Mini Kit (Qiagen). Libraries for RRBS were prepared according to the protocol previously described by Boyle et al [65]. For the MspI digestion 160 ng DNA was used. After end-repair and A-tailing, sample specific adaptors, with a unique sequence barcode for each sample, were ligated and purified by Agencourt AMPure XP beads (Beckman Coulter). The adaptor ligand mix was amplified by PCR (12 cycles) and purified by AMPure beads. All barcoded DNA samples were pooled into one library per donor, with respectively 7 DNA samples. The multiplexed libraries were amplified by PCR (12 cycles), purified by AMPure beads and sequenced (∼2.5 lanes per library) on the Illumina HiSeq 2500 platform in a 100 bp single read configuration. Since RRBS libraries exhibit low complexity base composition at the first three bases (due to the MspI digestion all fragments start with “CGG”) a tailored sequencing recipe with three initial dark cycles (designed with the help of Illumina) was used for the sequencing run. Raw reads were trimmed in RRBS-mode for adapter contamination and low-quality tails (Phred <20) with the Cutadapt wrapper Trim Galore! (http://www.bioinformatics.babraham.ac.uk/projects/trim_galore/) [66] for RRBS and mapped with GSNAP [67] to the human genome (hs37d5). After the initial alignment, Bis-SNP [68] was applied to realign the reads overlapping known insertions/deletions as defined by dbSNP v138, to recalibrate quality scores and finally to call methylation levels. This was done with default parameters, except a maximal allowed coverage of 1000. The methylation calls were filtered with RnBeads [69], requiring a minimum coverage of 5 reads in each sample. A genome wide comparison was performed by principle component analysis (PCA) of mean methylation levels from two donors for each cell-type and site. The PCA revealed a clear separation of CD4^+^ T_H_1 and CD8^+^ T memory cells, naïve CD4^+^ and CD8^+^ T cells, and NK cells by the first two components, with explained proportions of variance of 48.15%, 23.51%, 11.03%, 6.78%, 5.64% and 4.90% for PC1 to PC6. Moreover, 1,000 most variant methylation sites of 30,000 randomly chosen sites, localized within the gene bodies and promotor regions (−2 kb TSS) were used for clustering analysis and visualization of the methylation profiles (Beta-values of methylation sites) and similarities between cell subsets (Euclidian distance). This strategy was chosen to account for a representative proportion of genome wide methylation and to avoid a potential bias towards outstanding differences between particular cell types. The clustering was performed with the WARD-linkage criterion and Euclidian distance as similarity metric.

### Luciferase reporter assay

DNA constructs containing regulatory sequences of the human *IFNG* locus (CNS1 and promoter) were generated with the Expand High Fidelity PCR System (Roche) and cloned upstream of the Firefly luciferase (*Luc*) gene into the basic pGL3 reporter vector (Promega). For in vitro methylation assays, constructs were cloned into the CpG-free luciferase vector pCpGL (kindly provided by M. Rehli) [48]. pCpGL vectors were methylated in vitro by M.SssI according to manufacturer's recommendations (New England Biolabs), followed by purification with the NucleoSpin Extract II kit (Macherey Nagel). Transfection of rested (deprived of IL-2 for 24 hours) NKL cells was performed by nucleofection using the Amaxa Human NK cell Nucleofector Kit (Lonza). 3×10^6^ cells were transfected with 5 µg pGL3/pCpGL vector and 0.25 µg pRL-TK Renilla luciferase reporter vector (Promega), as internal control. After transfection, cells were rested for 1 hour followed by stimulation via NKG2C and/or 2B4 cross-linking as described before. Subsequently, cells were collected for measurement of luciferase activity, which was performed using the Dual Luciferase Reporter Assay system according to manufacturer's instructions (Promega) and analyzed with an Orion L luminometer (Titertek Berthold). Relative luciferase units (RLU) were normalized to Renilla luciferase and to empty vectors pGL3 or pCpGL, respectively. A list of all primers synthesized upon request (TIB Molbiol Berlin) is provided in Supporting Information.

### Statistical analysis

Wilcoxon signed rank two-tailed and Mann-Whitney test were used for statistical analysis of data sets consisting of at least six independent experiments (*p<0.05, **p<0.01, ***p<0.001), using GraphPad Prism (GraphPad Software).

## Supporting Information

Figure S1 **NKG2C expression in HCMV^+^ donors with or without expanded NKG2C^hi^ population.** NKG2C surface expression was analyzed by FC in NK cells derived from HCMV^+^ donors with or without expansion of NKG2C^hi^ NK cells (n = 10), gated on CD56^dim^ CD57^+^. NKG2C geometrical mean fluorescence intensity (MFI) is depicted as ratio of NKG2C^+/hi^/NKG2C^−^. *p<0.05, calculated with Mann-Whitney test.
(TIF)

Figure S2 ***IFNG*** **promoter demethylation is stably imprinted in NKG2C^hi^ memory-like NK cells.** CpG methylation analysis of the *IFNG* promoter performed in ex vivo FACS sorted CD56^dim^ CD57^+^ sKIR^+^ NKG2C^hi/−^ NK cell subsets derived from the HCMV^+^ donor depicted in Figure 3C. The same HCMV^+^ individual was analyzed twice with an interval of one year between the two measurements. CpG methylation of the *IFNG* promoter is depicted as mean percentage of methylation at each CpG site.
(TIF)

Figure S3 **Stimulation of expanded NKG2C^hi^ NK cells.** (A) Analysis of intracellular IFN-γ expression by FC after stimulation with IL-12 and IL-18 for 16 hours. Mean percentage of IFN-γ producing cells ± SEM is depicted for the indicated NK cell subsets. *p<0.05, calculated with Wilcoxon signed rank test. (B) *IL12RB2* mRNA expression (left) was detected by qPCR in NK cell subsets, which were FACS sorted as CD3^−^ CD56^dim^ CD57^+/−^ (CD62L^+/−^) NKG2C^+/−^ cells. mRNA expression is shown relative to CD56^dim^ CD57^−^ NKG2C^−^ NK cells after normalizing to *GAPDH*. Surface expression of IL-18Ra (right) was measured by FC in PBMC, after gating on CD3^−^ CD56^dim^ CD57^+/−^ (CD62L^+/−^) NKG2C^+/−^ NK cell subsets, with isotype control (solid grey histogram). One representative staining (n = 2) is shown. (C) Surface expression of NKp30 and NKp46 was measured by FC in PBMCs, after gating on CD3^−^ CD56^dim^ CD57^+/−^ (CD62L^+/−^) NKG2C^+/−^ cell subsets, with isotype control (solid grey histogram). One representative staining out of two is shown. (D and E) Viable CD3^−^ CD56^dim^ NK cells were FACS sorted and stimulated as indicated in Figure 5A and 5B. (D) Gating strategy to analyze intracellular IFN-γ expression of cells enriched in expanded NKG2C^hi^ NK cells in HCMV^+^ donors. Cells were gated being CD56^dim^ NKG2A^−^ CD57^+^, followed by gating on sKIR expression (KIR2DL3 in HLA-C1^+^ donor). One representative gating is depicted (n = 7). (E) Analysis of intracellular IFN-γ expression after cross-linking of NKp30 or NKp46 by NKG2C^hi^ expanded NK cells, gated as described in Figure S3D. One representative experiment out of two is depicted.
(TIF)

Figure S4 **NKL cells cultured in the presence of AZA.** (A–B) NKL cells were cultured for 5 days with 5 µM AZA or left untreated. (A) CpG methylation of the *IFNG* CNS1 (as described in Figure 1) was analyzed in NKL cells treated or not with AZA and is depicted as mean percentage of methylation at each CpG site. (B) Analysis of intracellular IFN-γ expression was performed by FC upon stimulation for 16 hours with aNKG2C alone or aNKG2C+a2B4. One representative experiment out of three is depicted.
(TIF)

Table S1 **List of primers used for cloning into Luciferase reporter vectors pGL3/pCpGL.**
(DOC)
