# Supplementary figures and images for: Human Cytomegalovirus Drives Epigenetic Imprinting of the IFNG Locus in NKG2Chi Natural Killer Cells
Source: PLoS Pathog. 2014 Oct 16;10(10):e1004441. doi: 10.1371/journal.ppat.1004441 (PMC4199780; doi:10.1371/journal.ppat.1004441)

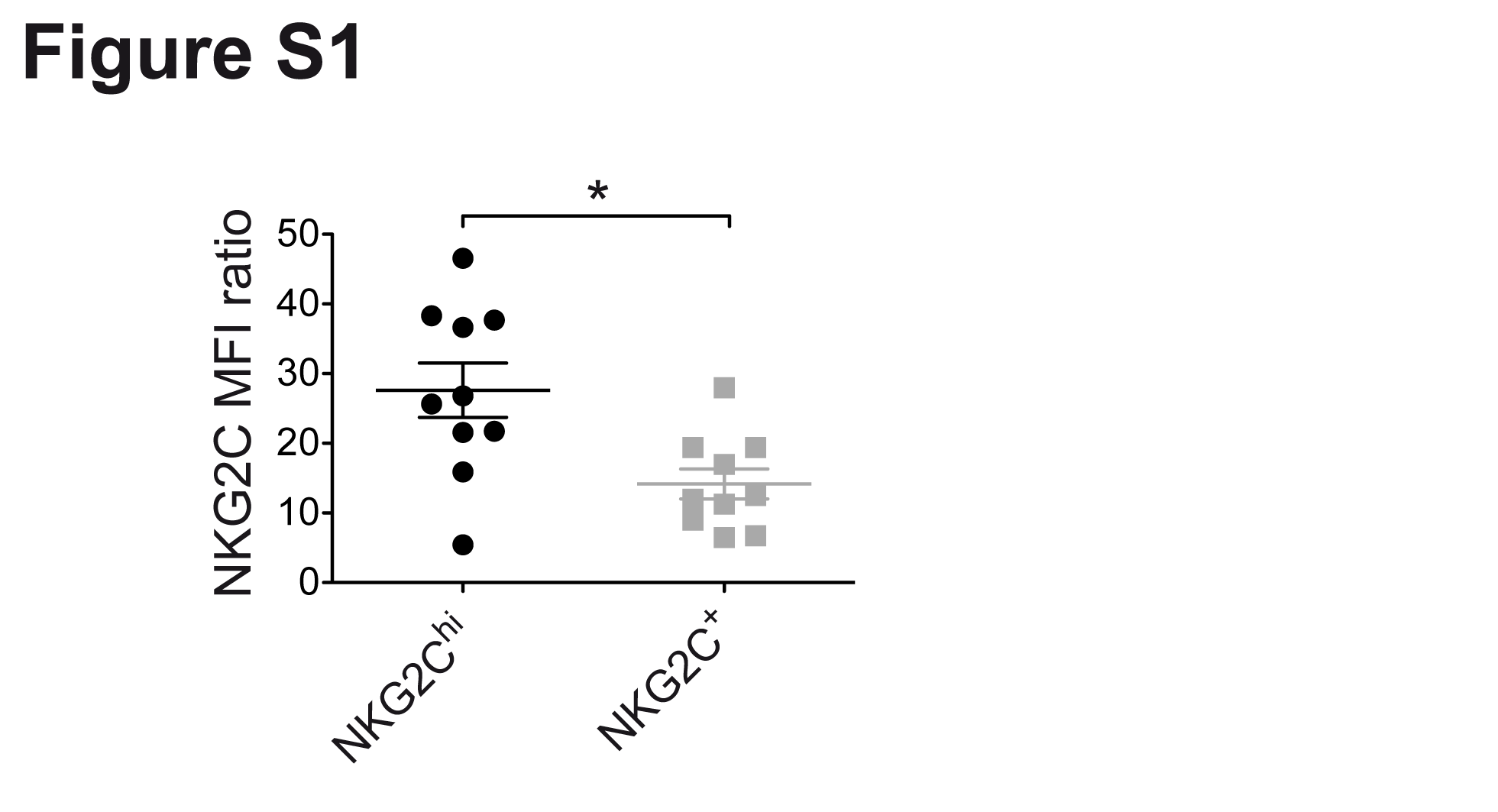

Supplement: Figure S1 — NKG2C expression in HCMV+ donors with or without expanded NKG2Chi population. NKG2C surface expression was analyzed by FC in NK cells derived from HCMV+ donors with or without expansion of NKG2Chi NK cells (n = 10), gated on CD56dim CD57+. NKG2C geometrical mean fluorescence intensity (MFI) is depicted as ratio of NKG2C+/hi/NKG2C−. *p<0.05, calculated with Mann-Whitney test. (TIF) [file ppat.1004441.s001.tif]

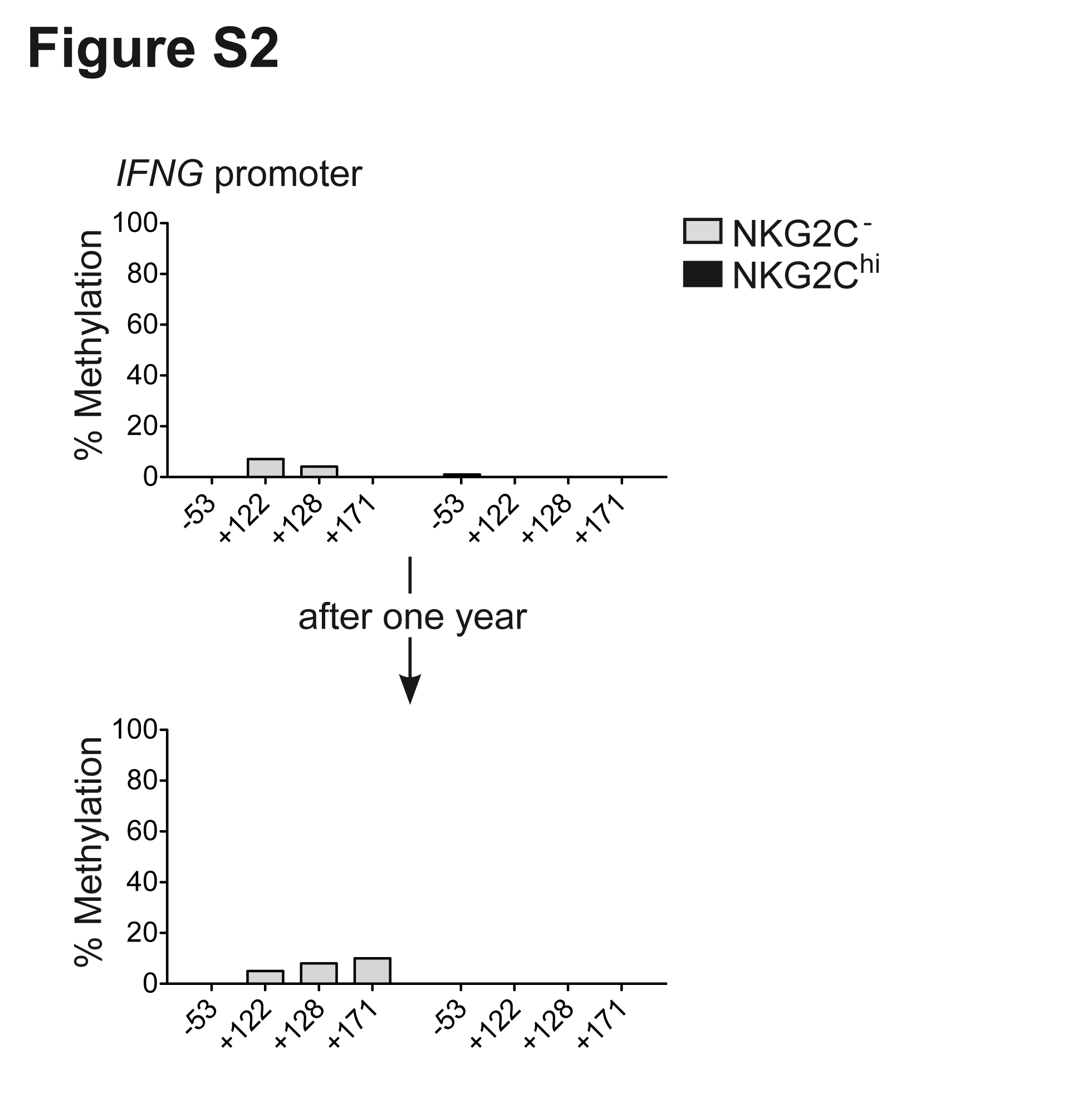

Supplement: Figure S2 — IFNG promoter demethylation is stably imprinted in NKG2Chi memory-like NK cells. CpG methylation analysis of the IFNG promoter performed in ex vivo FACS sorted CD56dim CD57+ sKIR+ NKG2Chi/− NK cell subsets derived from the HCMV+ donor depicted in Figure 3C. The same HCMV+ individual was analyzed twice with an interval of one year between the two measurements. CpG methylation of the IFNG promoter is depicted as mean percentage of methylation at each CpG site. (TIF) [file ppat.1004441.s002.tif]

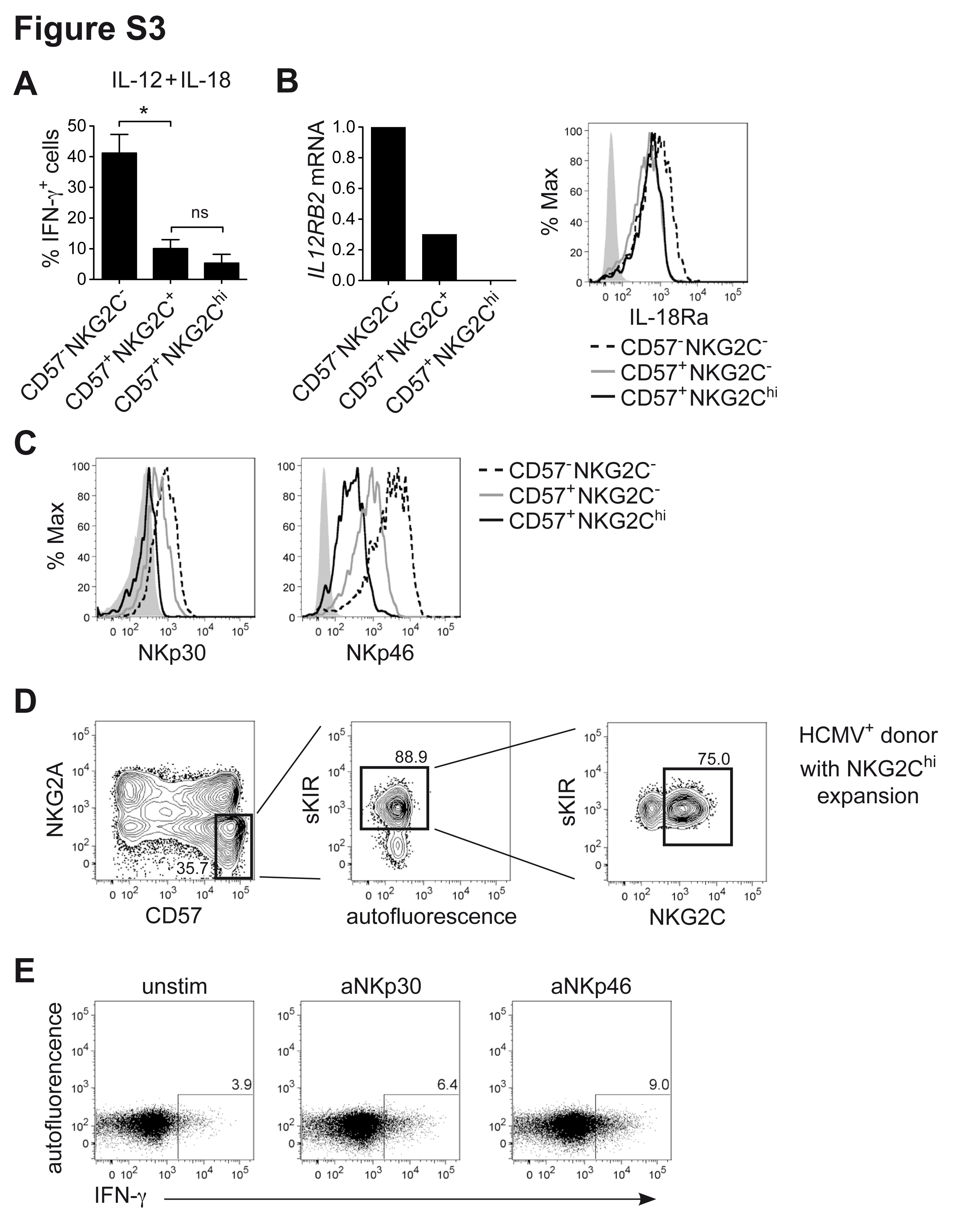

Supplement: Figure S3 — Stimulation of expanded NKG2Chi NK cells. (A) Analysis of intracellular IFN-γ expression by FC after stimulation with IL-12 and IL-18 for 16 hours. Mean percentage of IFN-γ producing cells ± SEM is depicted for the indicated NK cell subsets. *p<0.05, calculated with Wilcoxon signed rank test. (B) IL12RB2 mRNA expression (left) was detected by qPCR in NK cell subsets, which were FACS sorted as CD3− CD56dim CD57+/− (CD62L+/−) NKG2C+/− cells. mRNA expression is shown relative to CD56dim CD57− NKG2C− NK cells after normalizing to GAPDH. Surface expression of IL-18Ra (right) was measured by FC in PBMC, after gating on CD3− CD56dim CD57+/− (CD62L+/−) NKG2C+/− NK cell subsets, with isotype control (solid grey histogram). One representative staining (n = 2) is shown. (C) Surface expression of NKp30 and NKp46 was measured by FC in PBMCs, after gating on CD3− CD56dim CD57+/− (CD62L+/−) NKG2C+/− cell subsets, with isotype control (solid grey histogram). One representative staining out of two is shown. (D and E) Viable CD3− CD56dim NK cells were FACS sorted and stimulated as indicated in Figure 5A and 5B. (D) Gating strategy to analyze intracellular IFN-γ expression of cells enriched in expanded NKG2Chi NK cells in HCMV+ donors. Cells were gated being CD56dim NKG2A− CD57+, followed by gating on sKIR expression (KIR2DL3 in HLA-C1+ donor). One representative gating is depicted (n = 7). (E) Analysis of intracellular IFN-γ expression after cross-linking of NKp30 or NKp46 by NKG2Chi expanded NK cells, gated as described in Figure S3D. One representative experiment out of two is depicted. (TIF) [file ppat.1004441.s003.tif]

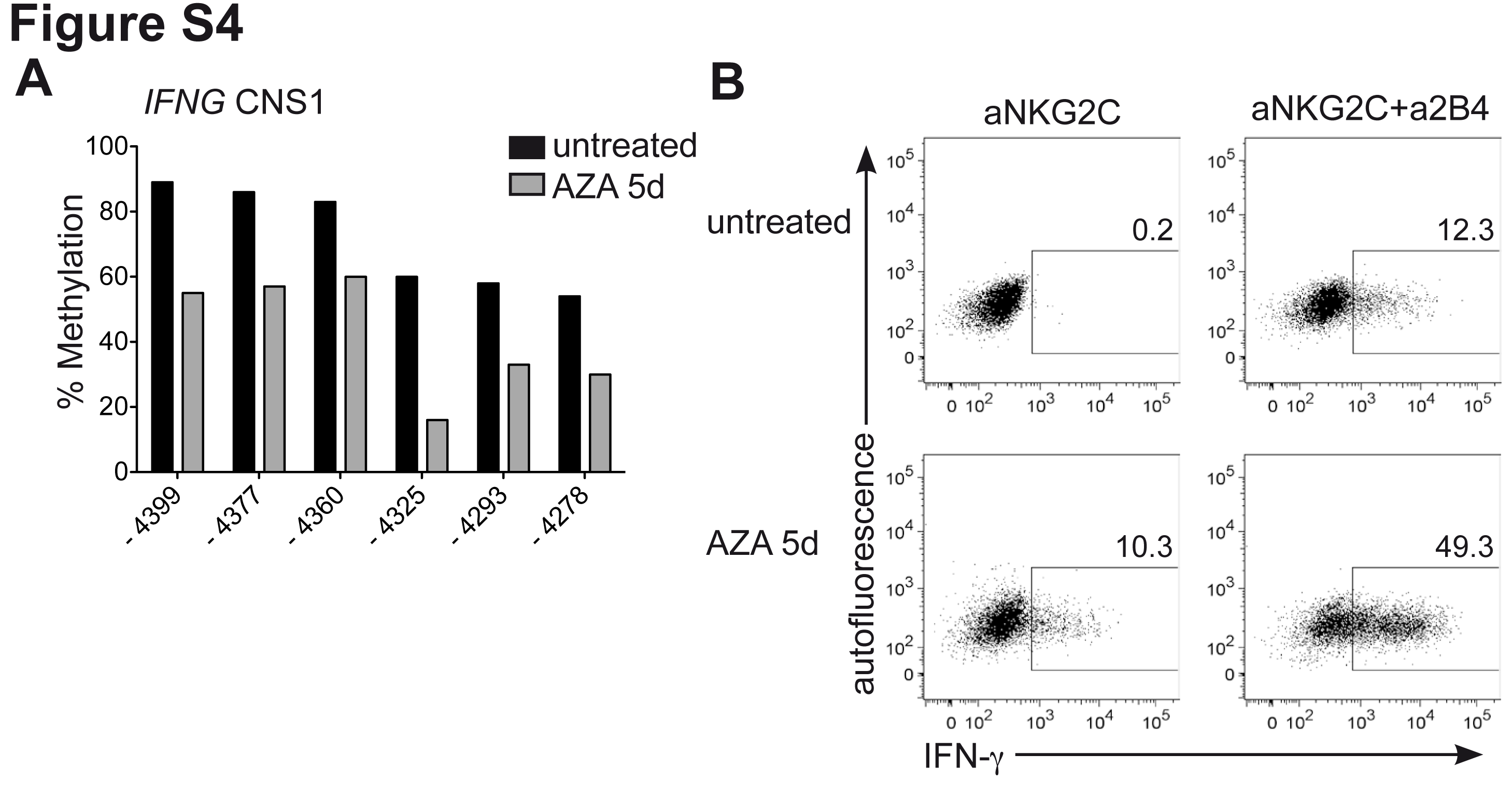

Supplement: Figure S4 — NKL cells cultured in the presence of AZA. (A–B) NKL cells were cultured for 5 days with 5 µM AZA or left untreated. (A) CpG methylation of the IFNG CNS1 (as described in Figure 1) was analyzed in NKL cells treated or not with AZA and is depicted as mean percentage of methylation at each CpG site. (B) Analysis of intracellular IFN-γ expression was performed by FC upon stimulation for 16 hours with aNKG2C alone or aNKG2C+a2B4. One representative experiment out of three is depicted. (TIF) [file ppat.1004441.s004.tif]
