# Supplementary material for: Human Cytomegalovirus Drives Epigenetic Imprinting of the IFNG Locus in NKG2Chi Natural Killer Cells
Source: PLoS Pathog. 2014 Oct 16;10(10):e1004441. doi: 10.1371/journal.ppat.1004441 (PMC4199780; doi:10.1371/journal.ppat.1004441)
Supplement: Table S1 — List of primers used for cloning into Luciferase reporter vectors pGL3/pCpGL. (DOC) [file ppat.1004441.s005.doc]

**Table S1: List of primers used for cloning into pGL3/pCpGL**

*Primers used for constructs cloned into pGL3:*

IFNG-Prom_fw-571-MluI AGTACGCGTCCTGTGTGGCTTGTATTGTATTTC

IFNG-Prom_rev+71- HindIII AGTAAGCTTTCAGGTCCAAAGGACTTAACTGA

*Primer used for constructs cloned into pCpGL:*

IFNG-Prom_fw-49- BglII AGTAGATCTTCCTCAGGAGACTTCAATTAGGTA

IFNG-Prom_rev+71- HindIII AGTAAGCTTTCAGGTCCAAAGGACTTAACTGA

IFNG-CNS1_fw- PstI AGTCTGCAGGCCAGAAGGCACAAAAGAAA

IFNG-CNS1_rev- BamHI AGTGGATCCCCAAATTATCTCATCCAGACT
